# Food Addiction in Gambling Disorder: Frequency and Clinical Outcomes

**DOI:** 10.3389/fpsyg.2017.00473

**Published:** 2017-04-04

**Authors:** Susana Jiménez-Murcia, Roser Granero, Ines Wolz, Marta Baño, Gemma Mestre-Bach, Trevor Steward, Zaida Agüera, Anke Hinney, Carlos Diéguez, Felipe F. Casanueva, Ashley N. Gearhardt, Anders Hakansson, José M. Menchón, Fernando Fernández-Aranda

**Affiliations:** ^1^Pathological Gambling Unit, Department of Psychiatry, Bellvitge University Hospital-IDIBELLBarcelona, Spain; ^2^Ciber Fisiopatología Obesidad y Nutrición (CIBERObn), Instituto de Salud Carlos IIIBarcelona, Spain; ^3^Department of Clinical Sciences, Faculty of Medicine, University of BarcelonaBarcelona, Spain; ^4^Departament de Psicobiologia i Metodologia de les Ciències de la Salut, Universitat Autònoma de BarcelonaBarcelona, Spain; ^5^Department of Child and Adolescent Psychiatry, University Hospital Essen, University of Duisburg-EssenEssen, Germany; ^6^Department of Physiology, Centro Singular de Investigación en Medicina Molecular y Enfermedades Crónicas, University of Santiago de Compostela-Instituto de Investigación SanitariaSantiago de Compostela, Spain; ^7^Laboratory of Molecular and Cellular Endocrinology, Research Area, Complejo Hospitalario Universitario de Santiago de CompostelaSantiago de Compostela, Spain; ^8^Department of Psychology, University of MichiganAnn Arbor, MI, USA; ^9^Lund University, Faculty of Medicine, Department of Clinical Sciences Lund, PsychiatryLund, Sweden; ^10^Ciber Salud Mental (CIBERSAM), Instituto Carlos IIIBarcelona, Spain

**Keywords:** food addiction, gambling disorder, comorbidity, sex, personality

## Abstract

**Background:** The food addiction (FA) model is receiving increasing interest from the scientific community. Available empirical evidence suggests that this condition may play an important role in the development and course of physical and mental health conditions such as obesity, eating disorders, and other addictive behaviors. However, no epidemiological data exist on the comorbidity of FA and gambling disorder (GD), or on the phenotype for the co-occurrence of GD+FA.

**Objectives:** To determine the frequency of the comorbid condition GD+FA, to assess whether this comorbidity features a unique clinical profile compared to GD without FA, and to generate predictive models for the presence of FA in a GD sample.

**Method:** Data correspond to *N* = 458 treatment-seeking patients who met criteria for GD in a hospital unit specialized in behavioral addictions.

**Results:** Point prevalence for FA diagnosis was 9.2%. A higher ratio of FA was found in women (30.5%) compared to men (6.0%). Lower FA prevalence was associated with older age. Patients with high FA scores were characterized by worse psychological state, and the risk of a FA diagnosis was increased in patients with high scores in the personality traits harm avoidance and self-transcendence, and low scores in cooperativeness (*R*^2^ = 0.18).

**Conclusion:** The co-occurrence of FA in treatment-seeking GD patients is related to poorer emotional and psychological states. GD treatment interventions and related behavioral addictions should consider potential associations with problematic eating behavior and aim to include techniques that aid patients in better managing this behavior.

## Introduction

### Food addiction

The applicability of the criteria for substance dependence disorders in the Fourth Edition of the Diagnostic and Statistical Manual of Mental Disorders (Álvarez-Moya et al., 2010) to behavioral addictions, including overeating, was greatly disputed (Moreno and Tandon, 2011). The Fifth Edition of the DSM (APA, 2013) chose to merge the diagnostic criteria for abuse and dependence into a single category of “substance-related and addictive disorders,” which listed only gambling disorder as a behavioral addiction, arguing that additional research-based validation was required in order to determine the transferability of the new DSM-5 criteria to other addictive behaviors (Pai et al., 2014; Potenza, 2014). Scientific research on food addiction (FA) is still in its nascent stages and currently, no consensus exists regarding a precise operational definition of FA, although this term is commonly used in areas such as obesity, eating disorders, and behavioral addictions. Systematic clinical and translational studies are scarce in the literature and evidence for a substance-related addiction to the specific nutrients found in foods is poor (Ziauddeen and Fletcher, 2013; Meule and Gearhardt, 2014; Long et al., 2015). Some authors have consequently posited that the term “eating addiction” may more accurately describe the behavioral components of addictive-like eating behavior (Avena et al., 2011; Hebebranda et al., 2014) than FA.

As the term *behavioral addiction* implies a continued, persistent, excessive, impulsive, and uncontrollable involvement in an activity despite the negative consequences, definitions for FA should accordingly include the combination of both, “substance-related” and “behavioral addiction” concepts. Recent research supports the notion that hyper-palatable foods may have addictive potential in some individuals because the increased potency of certain nutrients (Meule, 2015) and palliative properties may provide a form of self-medication (Fortuna, 2012) or natural reward (Hoch et al., 2015). Comprehensive reviews on studies in human and animal samples have also recognized that problematic eating behavior (including FA) constitutes a multifactorial condition that can involve a combination of metabolic, genetic, environmental, psychological, and behavioral factors, and that eating can be regulated by factors unrelated to metabolic control, such as stress and emotions (Macht, 2008; Hildebrandt and Greif, 2013; Di Segni et al., 2014).

Other results obtained in animal and human research within the context of the effects of food intake on brain reward systems have revealed that palatable foods can mimic the neurophysiological and behavioral effects of addictive drugs (Albayrak et al., 2012; de Jong et al., 2012; García-García et al., 2014; Cenci et al., 2015; Karlsson et al., 2015; Ziauddeen et al., 2015). Alterations in neurotransmission as a consequence of the perpetual intake of highly palatable foods have also been reported in both animal models and individuals with excess weight (Baik, 2013; Mietlicki-Baase et al., 2013; D'Souza, 2015). Furthermore, the anorexigenic effects of leptin also seem attenuated in FA, potentially leading to weakened food-reward (Bowen et al., 2014). Finally, some reviews centered on the neurobiological basis of FA and binge eating suggest that compulsive-addictive food intake could be considered from an evolutionary perspective, underscoring the importance of motivational systems involved in adaptive patterns of food intake (Salamone and Correa, 2013; Davis, 2014). Other studies propose that FA may simply be a more acute form of binge eating disorder (Davis, 2013) or a valid phenotype of obesity (Davis et al., 2011).

A genetic overlap between non-substance- and substance-related addictions has been implied by formal genetic studies (Slutske et al., 2000, 2013; Eisen et al., 2001; Blanco et al., 2012; Slutske and Richmond-Rakerd, 2014). The first genome-wide association study (GWAS) for food addiction (determined by the modified Yale Food Addiction Scale; mYFAS) in 9,314 women of European descent revealed two loci with genome wide significance (*P* < 2.5 × 10^−8^). Additionally, the GWAS data implied an enrichment for gene members of the MAPK signaling pathway (*P* = 0.02). However, candidate SNPs or genes for drug addiction were not associated with food addiction (Cornelis et al., 2016). Recently the first GWAS for pathological gambling was performed on 445 cases and 986 controls (Lang et al., 2016). Although, genome-wide significant variants were not detected, some pathway analyses were significant. Additionally, the analysis of a genetic overlap between pathological gambling and alcohol dependence revealed, by polygenic risk score analysis of the alcohol dependence dataset, a one-sided nominally significant *P*-value in individuals with pathological gambling. A combined analysis of genetic data pertaining to food addiction and gambling disorder has not yet been published.

Prevalence estimates for FA in developed countries vary greatly, depending on the assessment tools employed and the type of sample studied (e.g., general population, obese, student, or clinical samples). A systematic review that meta-analyzed 25 studies (*n* = 196,211) obtained a weighted mean prevalence of FA equal to 19.9% (Pursey et al., 2014). Studies using obese samples have obtained point prevalence rates between 34% (Ceccarini et al., 2015) and 40% (Meule et al., 2014); for university student samples point-prevalence is around 11% (Obregón et al., 2015). Epidemiological research further shows, that FA is more prevalent in women (Fattore et al., 2014), middle-aged and older individuals (Bowen et al., 2014; Flint et al., 2014), overweight/obese patients (Meule, 2012; Pedram et al., 2013; Lee et al., 2014), and in people of Black or Hispanic ethnicity or low socioeconomic status (Berenson et al., 2015).

### Gambling disorder

Gambling disorder (GD) is the only non-substance behavioral addiction in the diagnostic category “substance-related and addictive disorders” in the DSM-5 (APA, 2013). It constitutes a mental health disorder characterized by persistent and recurrent problematic gambling behavior leading to clinically significant impairment or distress. Numerous studies have reported empirical evidence on the frequency of GD in different samples/populations, its main risk factors, clinical phenotype, and treatment outcomes (Johansson et al., 2009; Cowlishaw et al., 2012; Bartley and Bloch, 2013; Gowing et al., 2015; Hing et al., 2015; Moragas et al., 2015).

Systematic reviews confirm commonalties between GD and other behavioral addictions (including FA) in terms of neural and psychological underpinnings (Cenci et al., 2015; Engel and Cáceda, 2015; Yau and Potenza, 2015; Grant et al., 2016), particularly with regard to (a) cognitive dysfunction manifested in the form of impulsivity and compulsivity; (b) structural and functional abnormalities of networks involved in reward processing and top-down control; (c) alterations in neurochemical-neuroendocrine systems implicated in pathophysiology; (d) elevated personality traits scores in negative urgency, disinhibition and novelty seeking; and (e) familial diathesis.

Epidemiological research outlines that worldwide prevalence for GD in adult populations has significantly increased in recent years. A recently published meta-analysis reported estimated prevalence of lifetime GD ranging from 0.01 to 10.6%, across studies, with higher point values among younger age groups and males, and higher risk-vulnerabilities for groups with fixed incomes and limited prospects of future earnings (Subramaniam et al., 2015).

Regarding comorbidity between eating disorders and gambling disorder, a study with a sample of 1,681 consecutive treatment-seeking eating disorder patients (1,576 females and 105 males), found that the lifetime prevalence of GD was 1.49%, similar to rates found in the general population, which stands at 1.5% (Jimenez-Murcia et al., 2013; Gowing et al., 2015). However, when considering ED subtype, GD was highly associated with binge eating disorder (5.7%). On the other hand, GD was also found to be more frequent in men (16%) than in women (1.26%), as seen from studies conducted both in the general population (Bonnaire et al., 2016) and in clinical samples (Erbas and Buchner, 2012; Jiménez-Murcia et al., 2014).

Another study, in this case, carried out with psychiatric inpatients, GD prevalence was found to be 9% and only one patient had an eating disorder associated with GD (Aragay et al., 2012). Despite the low comorbidity between the two conditions, results radically differ when the gender is considered. The fact that ED are more common in women has resulted in an overrepresentation of this gender in the literature and many studies have opted to exclude men from their study samples for the sake of homogeneity. Therefore, awareness of comorbidity between these two conditions is low.

However, GD and FA phenotypes share many common features. Firstly, both psychiatric conditions could be considered as forming part of the impulse control disorder spectrum, with the most evident shared attribute being the impulsive/compulsive nature of the addictive behavior (Leeman and Potenza, 2012; Grant and Chamberlain, 2014; Di Nicola et al., 2015; Konkolý Thege et al., 2015). Other shared characteristics are the early onset of these problematic/excessive behaviors (Balogh et al., 2013), high exposure to adverse life events (Lee et al., 2012), personality traits characterized by high scores in impulsivity, high levels of emotional-psychological distress (Karim and Chaudhri, 2012), and difficulties in emotion regulation (Williams et al., 2012; Pivarunas and Conner, 2015).

### Aims

Despite the similarities between GD and FA, to our knowledge no empirical study has estimated the co-occurrence of FA in GD samples, or the potential effects of the presence of FA in treatment-seeking GD samples. The objectives of this study were therefore: (a) to screen for the epidemiological occurrence of FA in a clinical sample of treatment-seeking patients who meet DSM-5 criteria for GD; (b) to assess whether GD patients with FA exhibit more severe gambling disorder severity, more maladaptive personality profiles, and greater general psychopathology, when compared to GD patients without FA; (c) to obtain predictive models of FA symptoms in patients with GD; and (d) to conduct a path analysis to explore the underlying mechanisms of GD and FA severity while considering patients' sex, age, and personality profile.

## Materials and methods

### Participants

Participants considered for the study were all patients referred to the Pathological Gambling Unit in the Psychiatry Department at Bellvitge University Hospital (Barcelona, Spain), for treatment of behavioral-addiction problems between September 2013 and December 2015 that met *DSM*-IV criteria for GD (called pathological gambling before the publication of the DSM-5) (*N* = 458). Bellvitge University Hospital is a public hospital certified as a tertiary care center for the treatment of addictive behaviors that oversees the treatment of highly complex cases. The catchment area of the hospital includes over two million people in Barcelona metropolitan area. All individuals who arrived to the specialized unit were assessed by expert clinical psychologists and psychiatrists with more than 15 years of clinical experience. Descriptive information for the total sample is included in Table 1. Most participants were male (87.1%), born in Spain (98.9%), with a primary (57.2%) or secondary school (35.8%) level of education, about half of the patients were married (49.1%). Mean age for the whole sample was 42.7 years (*SD* = 14.1), the mean age of onset of GD was 37.8 years (*SD* = 14.9) and the mean duration of problem gambling was 5.4 years (*SD* = 6.9).

**Table 1:** **Descriptives for the sample**.

		**Total; *n* = 458**		**Only-GD; *n* = 416**		**GD+FA; *n* = 42**				
		***n***	***%***	***n***	***%***	***n***	***%***	**χ^2^**	***df***	***p***
Sex	Female	59	12.9	41	9.9	18	42.9	37.02	1	<0.001^*^
	Male	399	87.1	375	90.1	24	57.1			
Origin	Spain	453	98.9	411	98.8	42	100	0.51	1	0.475
	Immigrant	5	1.1	5	1.2	0	0			
Education level	Primary	262	57.2	237	57.0	25	59.5	2.32	2	0.313
	Secondary	164	35.8	152	36.5	12	28.6			
	University	32	7.0	27	6.5	5	11.9			
Civil status	Single	175	38.2	154	37.0	21	50.0	2.75	2	0.253
	Married—in couple	225	49.1	208	50.0	17	40.5			
	Divorced—separated	58	12.7	54	13.0	4	9.5			
Employment status	Employed	231	51.0	206	50.1	25	59.5	1.35	1	0.246
Tobacco use	Yes	247	53.9	231	55.5	16	38.1	4.67	1	0.031^*^
Alcohol abuse	Yes	73	16.0	69	16.6	4	9.5	1.43	1	0.231
Other drug abuse	Yes	56	12.4	51	12.4	5	12.2	0.01	1	0.968
		**Mean**	***SD***	**Mean**	***SD***	**Mean**	***SD***	**T**	***df***	***p***
	Age (years)	42.67	14.06	43.12	14.00	38.17	14.05	2.18	456	0.029^*^
	Onset of GD (years)	37.81	14.88	38.21	14.92	33.90	14.02	1.79	456	0.074
	Duration of GD (years)	5.35	6.94	5.41	7.04	4.77	5.96	0.57	456	0.568

SD, standard deviation; GD, gambling disorder; FA, food addiction;

* *Bold: significant result (0.05 level)*.

### Instruments

#### Symptom checklist-revised (SCL-90-R; Derogatis, 1990)

The SCL-90 is a 90-item self-report questionnaire measured on an ordinal 3-point scale to evaluate a broad range of psychological problems and psychopathological symptoms. It is structured in nine primary symptom-dimensions: somatization, obsession-compulsion, interpersonal sensitivity, depression, anxiety, hostility, phobic anxiety, paranoid ideation, and psychoticism. Three global indices are also available: global severity index (GSI, a measure of overall psychological distress), positive symptom distress index (PSDI, a measure of the symptoms' intensity), and positive symptom total (PST, which reflects the total of self-reported symptoms). The Spanish adapted version was used in this study (Derogatis, 2002). Cronbach's alpha (α) in the sample of this study ranged from good to excellent (see α-values in **Table 3**).

#### Temperament and character inventory-revised (TCI-R; Cloninger, 1999)

Self-report to evaluate personality traits on 240-items measured on a 5-point Likert-type scale. It is structured in seven primary personality dimensions: four temperamental factors (novelty seeking, harm avoidance, reward dependence, and persistence) and three character dimensions (self-directedness, cooperativeness, and self-transcendence). The Spanish revised version used in this study (Gutiérrez-Zotes et al., 2004) showed adequate internal consistency (Cronbach's alpha α mean value of 0.87). Cronbach's alpha in the sample of this work was in the range moderate to excellent (see Table 3).

#### Diagnostic questionnaire for pathological gambling according to DSM criteria (Stinchfield, 2003)

This 19-item questionnaire allows assessing the DSM-IV (Álvarez-Moya et al., 2010) and DSM-5 (APA, 2013) diagnostic criteria for GD. Convergent validity with the SOGS scores in the original version was very good (*r* = 0.77 for representative samples and *r* = 0.75 for gambling treatment groups; (Stinchfield, 2003). Internal consistency of the Spanish adaptation used in this study was α = 0.81 for the general population and α = 0.77 for gambling treatment samples (Jiménez-Murcia et al., 2009b). In this study, the total number of DSM-5 criteria for GD was analyzed. α-value in the sample of this study was adequate (see Table 3).

#### South oaks gambling screen (SOGS; Lesieur and Blume, 1987)

Self-report 20-item screening questionnaire that discriminates between probable pathological, problem and non-problem gamblers. The Spanish validation used in this work showed excellent internal consistency (α = 0.94) and test-retest reliability (*r* = 0.98; Echeburúa et al., 1994). α-value in the study sample was adequate (see Table 3).

#### Yale food addiction scale (YFAS; Gearhardt et al., 2009)

This is a 25-item self-report questionnaire for measuring FA during the previous 12 months according to the seven symptoms of substance-dependence listed in the DSM-IV (APA, 2000). This instrument has been modified for eating behaviors and obtains two scores: (a) a quantitative dimensional score obtained as the sum of DSM-IV addictive symptoms (raw scores ranging from 0 to 7); and (b) a screening of FA diagnosis. A raw score higher than 3 combined with clinically significant impairment/distress is considered as meeting the criteria for FA diagnosis. The validation of the English version showed adequate internal consistency, good convergent, and incremental validity in predicting binge eating (Gearhardt et al., 2009). The Spanish version of the scale has also reported good psychometrical properties in Spanish-speaking samples with eating disorders (Granero et al., 2014) and internal consistency in this study sample was excellent (α = 0.93).

#### Additional data

Demographic, clinical, drug/alcohol, tobacco, and social/family variables were taken using a semi-structured face-to-face clinical interview (Jiménez-Murcia et al., 2006).

### Procedure

The present study was carried out in accordance with the latest version of the Declaration of Helsinki. The University Hospital of Bellvitge Ethics Committee of Clinical Research approved the study, and signed informed consent was obtained from all participants. Experienced psychologists and psychiatrists conducted two face-to-face clinical interviews, before and after the evaluation, in order to obtain clinical information that allows for an accurate diagnosis and that lets the clinicians choose the most appropriate treatment.

### Statistical analysis

Statistical analysis was carried out with Stata13.1 for Windows (StataCorp., 2013). Firstly, the initial sample of *N* = 458 participants was classified in two groups according to their FA diagnosis: GD without meeting FA diagnostic criteria on the YFAS (<3 criteria fulfilled; named only-GD in this work; *n* = 416) and GD with FA diagnosis on the YFAS (≥3 criteria fulfilled and clinically significant impairment/distress; named GD+FA in this work; *n* = 44). Analysis of Variance (ANOVA) procedures, adjusted for the covariates patients' sex and age, were used to compare the means in the quantitative clinical measures (gambling related variables, SOGS total score, SCL-90R, and TCI-R scale scores) between the only-GD and GD+FA groups. Bonferroni-Simes correction controlled the inflation in Type-I error due to multiple statistical comparisons (Simes, 1986). Effect sizes for the proportion and mean comparisons was estimated through Cohen's-*d* coefficient, considering |*d*| > 0.50 as a moderate effect size and |*d*| > 0.80 as a large effect size.

Secondly, partial correlations (also adjusted for the covariates sex and age) estimated the association between FA severity (dimensional YFAS raw scores) and clinical measures related to gambling, general psychopathology, and personality. |*r*| > 0.30 was considered good effect size.

Thirdly, step-wise regressions were used to estimate the best predictive models for the FA measure. Linear regression was used for the criterion YFAS raw total score, and adjusted-*R*^2^ measured the global predictive capacity of the final model. Logistic regression was used for the criterion of FA diagnosis on the YFAS scale (1 = present vs. 0 = absent). For the logistic model, Hosmer–Lemeshow test valued the goodness-of-fit of the final regression, Nagelkerke's *R*^2^ measured global predictive capacity and the area under the ROC curve (AUC) valued discriminative capacity. Modeling was done in two steps-blocks: the first block included and fixed the variables patients' sex and age, and the second block added and automatically selected the best predictors between the personality traits scores (TCI-R scales).

Finally, Structural Equation Modeling (SEM) was conducted to test the potential underlying mechanism through pathway analysis between patients' sex and age, personality traits, FA severity, and gambling related measures. The Maximum Likelihood method of parameter estimation was used and goodness-of-fit was evaluated using the usual statistics: the chi-square test (χ^2^), the Root Mean Square Error of Approximation (RMSEA), the Bentler's comparative Fit Index (CFI), the Tucker-Lewis Index (TLI), and the Standardized Root Mean Square Residual (SRMR). Adequate model fit was considered for non-significant χ^2^ test, RMSEA <0.08, TLI > 0.9, CFI > 0.9, and SRMR <0.1. The global predictive capacity of the model was measured with the Coefficient of Determination (CD).

## Results

### Epidemiology of GD+FA comorbidity

Table 2 contains the epidemiological indexes for the occurrence of FA measured through the YFAS questionnaire: the scores for the seven criteria for FA, the prevalence for the presence of impairment/distress due to FA, the prevalence of FA diagnosis, and the mean for FA severity (dimensional YFAS raw total score). The frequency distributions of Table 2 are tabulated for the total sample and for the subsample of patients who were given a FA diagnosis.

**Table 2:** **Distribution of the food addiction measures (YFAS)**.

	**Total; *n* = 458**		**Women; *n* = 59**		**Men; *n* = 399**					**Only-GD *n* = 416**		**GD+FA *n* = 42**				
	***n***	***%***	***n***	***%***	***n***	***%***	**χ^2^_*df* = 1_**	***p***	***|d|***	***n***	***%***	***n***	***%***	**χ^2^_*df* = 1_**	***p***	***|d|***
1. Long period	56	12.2	23	39.0	33	8.3	45.18	<0.001^*^	**0.78**^†^	33	7.9	23	54.8	77.95	<0.001^*^	**1.17**^†^
2. Persistent desire	419	91.5	58	98.3	361	90.5	4.04	0.044^*^	0.35	378	90.9	41	97.6	2.23	0.135	0.29
3. Much time	88	19.2	23	39.0	65	16.3	17.05	<0.001^*^	**0.52**^†^	53	12.7	35	83.3	122.5	<0.001^*^	**2.00**^†^
4. Social impairment	54	11.8	17	28.8	37	9.3	18.87	<0.001^*^	**0.51**^†^	33	7.9	21	50.0	64.91	<0.001^*^	**1.05**^†^
5. Use despite cons.	144	31.4	27	45.8	117	29.3	6.44	0.011^*^	0.34	117	28.1	27	64.3	23.14	<0.001^*^	**0.78**^†^
6. Tolerance	107	23.4	25	42.4	82	20.6	13.67	<0.001^*^	**0.50**^†^	77	18.5	30	71.4	59.67	<0.001^*^	**1.26**^†^
7. Withdrawal	52	11.4	20	33.9	32	8.0	34.20	<0.001^*^	**0.67**^†^	26	6.3	26	61.9	117.4	<0.001^*^	**1.45**^†^
Impairment-distress	51	11.1	19	32.2	32	8.0	30.38	<0.001^*^	**0.63**^†^	9	2.2	42	100.0	369.0	<0.001^*^	**9.51**^†^
FA: positive screen	42	9.2	18	30.5	24	6.0	37.02	<0.001^*^	**0.67**^†^	0	0	42	100	—	—	—
	**Mean**	***SD***	**Mean**	***SD***	**Mean**	***SD***	***F***_*df* = 1;456_	***p***	***|d|***	**Mean**	***SD***	**Mean**	***SD***	***F***_*df* = 1;456_	***P***	***|d|***
FA-raw-total score	2.01	1.49	3.27	2.19	1.82	1.25	54.44	<0.001^*^	**0.81**^†^	1.72	1.17	4.83	1.38	261.6	<0.001^*^	**2.44**^†^

*FA, food addiction; SD, standard deviation; |d|, Cohens'-d measuring effect size of differences*.

* *Bold: significant result (0.05 level)*.

† *Bold: moderate (|d| > 0.50) to high (|d| > 0.80) effect size*.

Considering the whole GD sample, the prevalence of patients with FA diagnosis was 9.17% (95%CI: 6.86–12.2%). Stratifying for the patients' sex, this prevalence was significantly higher for women (30.5%; 95%CI: 20.3–43.1%) than for men (6.02%; 95%CI: 4.08–8.79%) (χ^2^ = 19.1, *df* = 1, *p* < 0.001). Mean FA severity scores, measured through the dimensional YFAS raw total scores, also differed between genders (being higher for women than for men: 3.3 vs. 1.8; *F* = 54.4 *df* = 1–457, *p* < 0.001). Comparing each FA criterion and the presence of impairment/distress due to FA between genders, all items obtained higher prevalence for women than for men.

The comparison of each FA criterion between the two groups of the study (with and without a FA diagnosis) achieved significant results for all criteria except for “persistent desire.” Cohen's-*d* coefficients estimated high effect sizes for all criteria with significant results. These coefficients, which can also be interpreted as a measure of the item's relevance to differentiate between the groups, suggest that the most important discriminative criterion is the presence of impairment-distress, followed by *3-much time spent to obtain food, 7-withdrawal, 6-tolerance, 1-food consumed for long period/larger amount than intended, 4-social impairment*, and *5-use despite negative consequences*. Persistent desire was the least relevant criterion to differentiate between groups.

Table S1 contains the frequency distribution of the FA measures in the sub-sample GD+FA (*n* = 42), and the comparison between women and men. Point estimations showed that, as a whole, women had higher FA prevalence compared to men, but the two only criteria with significant differences between genders were *1- food consumed for long period/larger amount than intended* and *7-withdrawal*.

### Comparison between the only-GD and GD+FA diagnostic subtypes

Table 1 shows the comparison for the main socio demographic variables of the study. The percentage of women in the GD+FA group was statistically higher than for the GD-only condition (42.9 vs. 9.9%, *p* < 0.001). Statistical differences between diagnostic subtypes also emerged for tobacco use (higher prevalence in the only-GD group; 55.5 vs. 38.1%, *p* = 0.031) and age (higher mean for only-GD patients; 43.1 vs. 38.2 in the GD+FA group, *p* = 0.029). No differences emerged between the two groups for the age of onset and duration of gambling problems, the individuals' origin (Spanish nationals vs. those of non-community origin), education level, civil status, employment status, and drug use (alcohol and other substances).

The first part of Table 3 shows the results of the ANOVA adjusted for the patients' sex and age comparing the main clinical variables of the study between only-GD and GD+FA patients. The presence of high FA scores was statistically and clinically related to worse psychopathological states (higher means in all the SCL-90R scales), higher mean scores in the personality traits harm avoidance and self-transcendence, and lower means on the cooperativeness and self-directedness scales.

**Table 3:** **Association between clinical measures for patients with FA measures**.

		**Only-GD;** ***n*** = **416**		**GD**+**FA;** ***n*** = **42**		**ANOVA adjusted by sex-age**				**FA-raw-total score**^a^	
		**Mean**	***SD***	**Mean**	***SD***	**MD**	***F*_(1, 454)_**	***p***	***|d|***	***r***	***p***
Number addictive games		1.04	0.33	1.05	0.31	0.01	0.03	0.864	0.03	0.045	0.340
Maximum bets (euros)		2,301	16,629	1,005	4,095	12,95.2	0.23	0.633	0.11	0.088	0.059
Mean bets (euros)		181.08	940.32	56.59	127.87	124.49	0.66	0.417	0.19	−0.027	0.569
Cumulate debts (euros)		12,448	50,753	7,542	29,054	4906.1	0.34	0.558	0.12	−0.009	0.845
DSM-5: total criteria	α = 0.74	6.88	2.15	7.39	2.21	0.52	1.99	0.159	0.24	0.086	0.065
SOGS: total score	α = 0.73	10.00	3.15	10.43	3.36	0.43	0.64	0.425	0.13	0.034	0.463
SCL-90R: Somatization	α = 0.90	0.94	0.81	1.70	1.06	0.77	30.43	<0.001^*^	**0.81**^†^	0.263	<0.001^*^
SCL-90R: Obsessive/comp.	α = 0.87	1.13	0.83	1.84	1.06	0.70	24.53	<0.001^*^	**0.74**^†^	0.220	<0.001^*^
SCL-90R: Sensitivity	α = 0.87	1.05	0.85	1.72	1.08	0.67	21.06	<0.001^*^	**0.68**^†^	0.210	<0.001^*^
SCL-90R: Depressive	α = 0.91	1.55	0.95	2.18	1.08	0.64	15.89	<0.001^*^	**0.63**^†^	0.185	<0.001^*^
SCL-90R: Anxiety	α = 0.89	1.02	0.82	1.77	1.14	0.76	28.16	<0.001^*^	**0.76**^†^	0.255	<0.001^*^
SCL-90R: Hostility	α = 0.83	0.93	0.83	1.48	1.09	0.55	14.56	<0.001^*^	**0.57**^†^	0.157	0.001^*^
SCL-90R: Phobic anxiety	α = 0.83	0.46	0.66	1.10	1.19	0.64	27.24	<0.001^*^	**0.66**^†^	0.240	<0.001^*^
SCL-90R: Paranoid	α = 0.78	0.96	0.78	1.56	1.06	0.60	19.63	<0.001^*^	**0.65**^†^	0.214	<0.001^*^
SCL-90R: Psychotic	α = 0.84	0.90	0.77	1.53	0.89	0.63	22.56	<0.001^*^	**0.75**^†^	0.236	<0.001^*^
SCL-90R: GSI score	α = 0.98	1.07	0.71	1.74	0.94	0.68	30.13	<0.001^*^	**0.81**^†^	0.258	<0.001^*^
SCL-90R: PST score	α = 0.98	46.96	21.35	61.81	19.48	14.85	17.29	<0.001^*^	**0.73**^†^	0.188	<0.001^*^
SCL-90R: PSDI score	α = 0.98	1.87	0.59	2.34	0.71	0.47	21.96	<0.001^*^	**0.72**^†^	0.261	<0.001^*^
TCI-R: Novelty seeking	α = 0.73	108.89	14.68	109.99	13.43	1.10	0.20	0.762	0.08	0.088	0.060
TCI-R: Harm avoidance	α = 0.83	101.03	17.45	108.58	16.83	7.55	6.51	0.026^*^	**0.50**^†^	0.106	0.023^*^
TCI-R: Reward dependence	α = 0.77	99.37	14.82	99.96	12.93	0.59	0.06	0.812	0.04	−0.002	0.958
TCI-R: Persistence	α = 0.88	106.02	22.22	108.96	18.15	2.94	0.62	0.604	0.14	0.016	0.732
TCI-R: Self-directedness	α = 0.87	130.68	21.89	117.02	21.24	13.66	13.55	0.002^*^	**0.63**^†^	−0.216	<0.001^*^
TCI-R: Cooperativeness	α = 0.81	131.97	16.69	125.17	19.35	6.81	5.56	0.003^*^	0.38	−0.156	0.001^*^
TCI-R: Self-Transcendence	α = 0.84	62.38	14.91	70.31	15.10	7.93	10.38	0.005^*^	**0.53**^†^	0.182	<0.001^*^

a *Partial correlation adjusted by sex and age*.

*GD, gambling disorder; FA, food addiction; SOGS, South Oaks Gambling Screen; SCL-90R, Symptom Checklist-Revised; TCI-R, Temperament and Character Inventory—Revised*.

* *Bold: significant comparison (0.05 level)*.

† *Bold: moderate (|d| > 0.50) to high (|d| > 0.80) effect size. p-values include Bonferroni–Simes correction for multiple statistical tests*.

The second part of Table 3 contains partial correlations (also adjusted for patients' sex and age) between the dimensional FA-raw-total score and clinical measures. High FA scores were related to worse psychopathological state (higher SCL-90-R scores). Regarding personality traits, FA-raw-total scores were significantly and positively associated with harm avoidance and self-transcendence and negatively correlated with self-directedness and cooperativeness.

### Predictive model for FA diagnosis and severity

The first model shown in Table 4 corresponds to the final logistic regression measuring the contribution of sex and age to the presence of a FA diagnosis on the YFAS (1 = present vs. 0 = absent), and the main personality predictors of this criterion. Results indicate that risk of a FA diagnosis is higher for women; patients of a younger age and those with higher scores in the personality traits harm avoidance and self-transcendence. The predictive capacity of the final model was good (Nagelkerke's-*R*^2^ = 0.22) as well as its discriminative capacity (AUC = 0.86).

**Table 4:** **Predictive models for the outcomes FA diagnosis and FA total score**.

**Criterion: FA diagnosis**	**B**	**SE**	**Wald(1)**	***p***	**OR**	**95%CI (OR)**	
Sex (female)	1.799	0.378	22.625	<0.001	6.04	2.88	12.68
Age (years-old)	−0.035	0.014	6.590	0.010	0.97	0.94	0.99
TCI-R: Harm avoidance	0.028	0.010	7.325	0.007	1.03	1.01	1.05
TCI-R: Self-Transcendence	0.033	0.011	8.912	0.003	1.03	1.01	1.06
Constant	−6.436	1.477	18.975	<0.001	0.01		
Fitting: Hosmer-Lemeshow = 0.114; Nagelkerke's-*R*^2^ = 0.22; AUC = 0.86							
**Criterion: FA total score**	**B**	**SE**	**Beta**	***t***	***p***	**95%CI (B)**	
Sex (female)	1.335	0.193	0.301	6.929	<0.001	0.956	1.714
Age (years-old)	−0.015	0.005	−0.145	−3.302	0.001	−0.024	−0.006
TCI-R: Harm avoidance	0.007	0.004	0.087	2.010	0.045	0	0.015
TCI-R: Cooperativeness	−0.011	0.004	−0.130	−3.004	0.003	−0.019	−0.004
TCI-R: Self-Transcendence	0.018	0.004	0.178	4.027	<0.001	0.009	0.026
Constant	2.121	0.736		2.882	0.004	0.675	3.567
Fitting: Adjusted-*R*^2^ = 0.182							

*FA, food addiction; TCI-R, Temperament and Character Inventory—Revised; AUC, area under ROC curve*.

The second model shown in Table 4 corresponds to the final multiple linear regression measuring the contribution of sex and age on the dimensional YFAS-raw-total score (measuring FA severity), and the main personality predictors of this criterion. This model indicated that FA severity was higher for women, patients of a younger age and higher scores in the personality traits harm avoidance and self-transcendence, and lower scores in cooperativeness. The predictive capacity of the final model was good (Nagelkerke's-*R*^2^ = 0.18).

### SEM exploring the interrelationships between sex, age, personality, FA, and gambling

**Figure 1:**
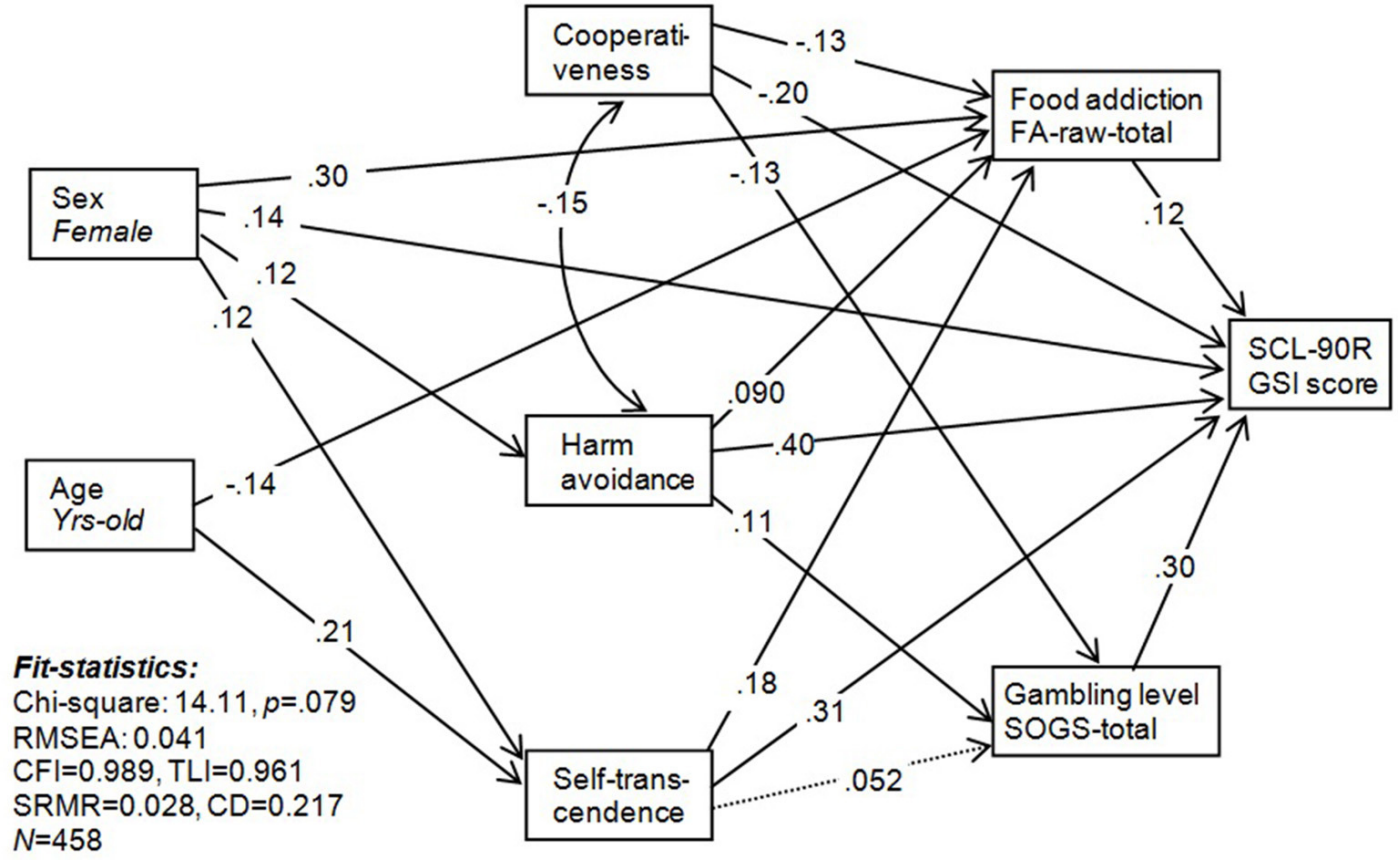
**SEM for the pathways between sex, age, personality traits, food addiction, and gambling**.

Figure 1 contains the pathway analysis with the main variables of the study explaining FA and GD severity. Results confirm the direct associations obtained in the previous regression models: FA severity is explained by being female, younger age, higher scores in the personality traits harm avoidance and self-transcendence, and lower scores in cooperativeness. And in addition to these direct associations, two relevant mediation effects also emerged: (a) FA severity was a mediating factor in the relationships between patients' sex, age, and the three personality traits on the one hand, and global psychopathological state on the other hand (SCL-90-R GSI score); (b) gambling severity (SOGS-total score) was a mediator between the personality traits cooperativeness and harm avoidance and psychopathological state (SCL-90-R GSI). Other mediation effects were found for the personality traits scores: harm avoidance mediated the relationships between sex and FA severity, sex and gambling severity, and sex and psychopathological state; and self-transcendence mediated the association between sex, age, and FA severity and psychopathological state. Goodness-of-fit was good for the final model, and the global predictive capacity was high.

## Discussion

This study analyzed the frequency of the co-occurrence of GD with FA, and the specific characteristics of this comorbidity compared to GD without FA. The prevalence of FA in the GD sample was 9.7%, with an increased ratio of women compared to men (31.3 vs. 6.9%) and decreasing prevalence at older ages. The comorbidity GD+FA is associated with worse global psychological state than GD only. The risk of obtaining a FA diagnosis was higher for women, patients with younger age and those with higher scores in the personality traits harm avoidance and self-transcendence. Similar results were obtained regarding the FA severity; in addition to the predictors previously described this model indicated an association between low levels of cooperativeness and FA in GD patients.

Scientific literature evidences that FA is more common in women (Pursey et al., 2014) and that it is associated with higher levels of negative affect and depression, and with higher general psychopathology (Granero et al., 2014). Few studies have analyzed the relationship between personality traits and the presence of FA conditions (Wolz et al., 2016) and, to the best of our knowledge, this is the first time that FA is assessed in a clinical GD sample. Personality traits commonly described in GD are high levels of novelty-seeking, low self-directedness and low cooperativeness (Janiri et al., 2007; Álvarez-Moya et al., 2010). Similarly, other studies have demonstrated the relationship between temperament traits like harm avoidance and GD (Nordin and Nylander, 2007; Moragas et al., 2015; Jimenez-Murcia et al., 2016). High levels of harm avoidance is characterized by introspective features and in GD patients, especially women, can lead to the use of gambling as a means of regulating negative affective states (Ledgerwood and Petry, 2006; Stewart and Zack, 2008; Smith et al., 2015; Jimenez-Murcia et al., 2016).

In the current study, when comparing GD+FA with only GD, results showed that mean levels of self-directedness were significantly lower in GD+FA patients. This is consistent with another study, conducted in eating disorder outpatients, showing that FA is strongly related to low self-directedness (Wolz et al., 2016). Moreover, self-directedness is a personality feature described extensively in both GD and other behavioral addictions (Granero et al., 2016a,b), as well as in eating disorders with and without associated behavioral addictions (Moragas et al., 2015). Apart from this, patients with FA were found to have higher scores in self-transcendence (individuals with this personality trait tend to be unconventional, illogical, suspicious, and immature; Cloninger et al., 1998). In this line, previous studies observed that high scores in self-transcendence were a clear predictor of both abuse of and/or dependence on alcohol and drugs, in a sample of GD outpatients (Jiménez-Murcia et al., 2009a). This finding was in agreement with those of other studies carried out in SUD patients (Simmons and Havens, 2007; Herrero et al., 2008). Furthermore, research aimed at the identification of distinct subtypes of GD patients described the existence of a subgroup denominated as “disorganized and emotionally unstable,” which is characterized by high impulsiveness and self-transcendence, substance and alcohol abuse and early age of onset as well as psychopathological disturbances. Interestingly, the presence of women was especially high in this subtype (Álvarez-Moya et al., 2010). In congruence with the results of the present study and the findings described above, Bégin et al. (2012) found that in three groups of women, two of them with overweight/obesity (one with and one without comorbid FA) and a third group with SUD, the groups with overweight/obesity + FA, and SUD were more similar, in terms of personality traits (impulsivity, personality, sensitivity to punishment, and reward, etc.), when compared to the third group with overweight/obesity, but without FA. However, it's worth noting that tobacco use was negatively associated with GD+FA, though this could be reflected by the fact that there were more women in this group.

In addition to the direct associations described above, our analysis has also shown a relationship between these variables (sex, age, and personality traits) and emotional distress (measured by the SCL-90-R).

This pathway suggests that in behavioral addictions, such as GD, there may be a differentiated phenotype of patients, especially young women, presenting addictive-like eating patterns in the context of emotion regulation problems. In fact, various studies conducted with samples of women with GD conclude that gambling is used as a maladaptive way to avoid feelings of frustration, sadness, isolation, and dissatisfaction with their lives (Martins et al., 2008; Fattore et al., 2014; Aymamí et al., 2015; Moragas et al., 2015). Other research identified a direct association between high levels of harm avoidance and psychopathology in women, suggesting that this population might be vulnerable to developing other comorbid disorders (Granero et al., 2009). Therefore, based on the results obtained in this research, it could be postulated that both behaviors (gambling and eating) are dysfunctional strategies that women with GD use to regulate negative emotional states. It is important to note that although FA has not yet been accepted in diagnostic manuals of mental disorders (as in the case of other excessive behaviors like shopping, gaming, etc.; Potenza, 2014) and although it is a controversial issue (Hebebranda et al., 2014; Wolz et al., 2016), the fact that a subgroup of GD patients (mostly women) in addition to their gambling problem suffers from FA demonstrates the importance of exploring the correlates of this condition (Gearhardt et al., 2009).

It is therefore advocated to systematically assess the existence of FA in patients with substance and behavioral addictions and to be especially aware in cases of young women who present overweight or obesity. From a therapeutic point of view, it is necessary to design and implement programs based on holistic interventions that address skills and techniques to improve the two conditions (as in GD with SUD, because of the high co-occurrence). In short, the most relevant issue is to offer problem-solving strategies to the patient, in order to improve self-control, mood state, and quality of life.

### Limitations

There are several methodological limitations to this study that need to be taken into account. First, the participants in the sample are only representative of GD patients who seek treatment and therefore the findings obtained may not apply to all individuals with GD. Since few GD individuals seek help for their disorder, a community sample of GD may yield different results. Second, the use of a standardized self-administered questionnaire as assessment procedure did not allow for an in-depth evaluation of specific Axis I and II comorbid disorders. Third, the cross-sectional nature of the study does not allow to conclude if the personality traits found to be related to FA precede or succeed FA symptoms, or if both have one common cause. Moreover, the present study only included one self-report measure of FA, which could be influenced by other variables related to this condition.

## Conclusion

In sum, the results of this study outline that the comorbid condition of GD with FA is related to a specific phenotype different to that obtained for GD patients without FA. Differences are especially evident for sex and age distribution, and for general psychopathology levels. As a whole, these findings highlight that GD constitutes a heterogeneous condition and that FA should be considered an identifiable and distinct clinical feature with specific clinical outcomes.

The concept of FA needs to be rethought and requires further research. Advanced empirical studies, addressing the etiology and development of FA, as well as to the co-occurrence of FA with other psychiatric mental conditions (such as GD), are needed. Research on neurochemical pathways (for example based on neurobiological models showing overlaps for chemical substances and behavioral addictions) could identify which specific brain regions (prefrontal areas, subcortical structures, and sensory areas) and neurotransmitter systems contribute to the course of non-homeostatic feeding and its association with other behavioral addictions. A better understanding of the mechanisms underlying the onset, clinical profile, and development of the GD+FA comorbidity will allow mental health preventive and intervention services to utilize precise routine assessment tools and adapted treatments for this specific addiction profile (Gearhardt and Corbin, 2011; Sauvaget et al., 2015).

## Author contributions

SJ, AG, and FF contributed to the development of the study conceptualization and design. RG performed the formal statistical analysis. MB, IW, GM, TS, and ZA conducted the research and investigation processes of this study, specially data collection. TS, GM, RG, FF, SJ, and AG aided with our interpretation of data. SJ, JM, and FF obtained funding. SJ, RG, AHi, FF, AHa, FC, and CD supervised the study. TS, RG, AG, FF, SJ, and GM were involved in the creation and writing of the initial draft.

## Funding

Financial support was received through the Ministerio de Economía y Competitividad grant (PSI2015-68701-R), FIS (PI14/00290) and cofunded by FEDER funds/European Regional Development Fund (ERDF), a way to build Europe, and AGAUR (2014 SGR 1672). CIBER Fisiología Obesidad y Nutrición (CIBERobn) and CIBER Salud Mental (CIBERSAM) are both initiatives of ISCIII. IW was supported by a predoctoral AGAUR grant (2016FI_B2 00001). GM was supported by a predoctoral AGAUR grant (2016FI_B 00568).

### Conflict of interest statement

The authors declare that the research was conducted in the absence of any commercial or financial relationships that could be construed as a potential conflict of interest.
