# Supplementary material for: Food Addiction in Gambling Disorder: Frequency and Clinical Outcomes
Source: Front Psychol. 2017 Apr 4;8:473. doi: 10.3389/fpsyg.2017.00473 (PMC5378803; doi:10.3389/fpsyg.2017.00473)
Supplement: Supplementary file 1 [file Table1.PDF]

Table S1 (supplementary)

Differential Distribution among sex of the food addiction measure (YFAS) in the sample with a FA diagnosis

	Total; *n*=42		Women; *n*=18		Men; *n*=24				
	n	%	*n*	%	*n*	%	$\chi^2_{df=1}$	*p*	|*d*|
1. Long period	23	54.8	14	77.8	9	37.5	6.74	**.009***	**0.89**†
2. Persistent desire	41	97.6	18	100	23	95.8	0.77	.381	0.29
3. Much time	35	83.3	14	77.8	21	87.5	0.70	.403	0.26
4. Social impairment	21	50.0	11	61.1	10	41.7	1.56	.212	0.40
5. Use despite cons.	27	64.3	13	72.2	14	58.3	0.86	.353	0.29
6. Tolerance	30	71.4	13	72.2	17	70.8	0.01	.921	0.03
7. Withdrawal	26	61.9	17	94.4	9	37.5	14.14	**<.001***	**1.50**†
Impairment-distress	42	100.0	18	100	24	100	---	---	---
	Mean	SD	Mean	SD	Mean	SD	$F_{df=1;41}$	*p*	|*d*|
FA-raw-total score	4.83	1.38	5.56	1.46	4.29	1.04	10.70	**.002**	**0.99**†

Note. FA: food addiction. SD: standard deviation. |*d*| Cohens'-*d* measuring effect size of differences.

*Bold: significant result (.05 level). †Bold: moderate (|*d*|>0.50) to high (|*d*|>0.80) effect size.
